# Quantitative chemical exchange saturation transfer imaging of nuclear overhauser effects in acute ischemic stroke

**DOI:** 10.1002/mrm.29187

**Published:** 2022-03-07

**Authors:** Yunus Msayib, George W. J. Harston, Kevin J. Ray, James R. Larkin, Brad A. Sutherland, Fintan Sheerin, Nicholas P. Blockley, Thomas W. Okell, Peter Jezzard, Andrew Baldwin, Nicola R. Sibson, James Kennedy, Michael A. Chappell

**Affiliations:** ^1^ Institute of Biomedical Engineering, Department of Engineering Science University of Oxford Oxford UK; ^2^ Acute Vascular Imaging Centre, Radcliffe Department of Medicine University of Oxford Oxford UK; ^3^ Wellcome Centre for Integrative Neuroimaging, Nuffield Department of Clinical Neurosciences University of Oxford Oxford UK; ^4^ Department of Oncology, CRUK and MRC Oxford Institute for Radiation Oncology University of Oxford Oxford UK; ^5^ School of Medicine University of Tasmania Hobart Tasmania Australia; ^6^ Department of Chemistry University of Oxford Oxford UK; ^7^ Sir Peter Mansfield Imaging Center, School of Medicine University of Nottingham Nottingham UK; ^8^ Mental Health & Clinical Neuroscience, School of Medicine, University of Nottingham Nottingham UK

**Keywords:** acute ischemic stroke, chemical exchange saturation transfer, nuclear Overhauser effect

## Abstract

**Purpose:**

In chemical exchange saturation transfer imaging, saturation effects between −2 to −5 ppm (nuclear Overhauser effects, NOEs) have been shown to exhibit contrast in preclinical stroke models. Our previous work on NOEs in human stroke used an analysis model that combined NOEs and semisolid MT; however their combination might feasibly have reduced sensitivity to changes in NOEs. The aim of this study was to explore the information a 4‐pool Bloch–McConnell model provides about the NOE contribution in ischemic stroke, contrasting that with an intentionally approximate 3‐pool model.

**Methods:**

MRI data from 12 patients presenting with ischemic stroke were retrospectively analyzed, as well as from six animals induced with an ischemic lesion. Two Bloch–McConnell models (4 pools, and a 3‐pool approximation) were compared for their ability to distinguish pathological tissue in acute stroke. The association of NOEs with pH was also explored, using pH phantoms that mimic the intracellular environment of naïve mouse brain.

**Results:**

The 4‐pool measure of NOEs exhibited a different association with tissue outcome compared to 3‐pool approximation in the ischemic core and in tissue that underwent delayed infarction. In the ischemic core, the 4‐pool measure was elevated in patient white matter (1.20±0.20) and in animals (1.27±0.20). In the naïve brain pH phantoms, significant positive correlation between the NOE and pH was observed.

**Conclusion:**

Associations of NOEs with tissue pathology were found using the 4‐pool metric that were not observed using the 3‐pool approximation. The 4‐pool model more adequately captured in vivo changes in NOEs and revealed trends depending on tissue pathology in stroke.

## 1 INTRODUCTION

Chemical exchange saturation transfer (CEST) MRI is a magnetization transfer (MT) imaging technique that employs the exchange of magnetization between low concentrations of endogenous or exogenous contrast species and the bulk water pool.^1^, ^2^ An endogenous contrast mechanism that can be imaged using CEST MRI is saturation transfer arising from nuclear Overhauser effects (NOEs). NOEs correspond to magnetization that is relayed through a combination of dipolar‐coupling from aliphatic to labile protons, before undergoing chemical exchange with the water pool.^3^ NOEs, if associated with cell metabolism, may help to identify the ischemic penumbra in stroke patients, the classical definition of which is a region of viable tissue around the ischemic core that is hypoperfused and metabolically stressed.^4^, ^5^, ^6^ The existing CEST literature is not conclusive on some of the characteristics of NOEs, including association with tissue pH and the mechanisms involved in transfer of magnetization.^3^, ^7^, ^8^, ^9^, ^10^

A 3‐pool Bloch–McConnell model has been used in our previous clinical CEST studies^11^, ^12^ to examine amide proton transfer (APT) effects. The three pools modeled a water pool and an amide pool, while also attempting to correct for spectral asymmetry using a third pool that combined upfield effects (mostly bracketed between 0 and −5 ppm) with spectrally broader MT effects. A clinical demonstration of changes in NOEs using the 3‐pool model‐based analysis in acute human stroke was reported by Tee et al.^13^ on the data from the study in Reference 11, finding a decrease in saturation effects at −2.41 ppm in the ischemic core. In the 3‐pool model, the combination of semisolid and NOE effects might feasibly have introduced bias into the measures of, or reduced the sensitivity to, changes in NOEs. The more recent literature suggests that semisolid MT and NOEs are distinct, and would not be modeled well as a combined effect.^7^, ^14^ It is apparent, therefore, that if NOEs are to be explored as a phenomenon in their own right in stroke, it will be necessary to use an analysis model that isolates them from contaminating exchange processes associated with semisolid MT. At least four pools might, therefore, be necessary in order to separately quantify NOEs, semisolid MT effects, and APT. We have previously examined the use of a 4‐pool model in the context of acute stroke data for APT quantification, finding that it was more robust than nonmodel‐based approaches that seek to separate upfield and downfield CEST effects.^15^ The 4‐pool model was also more robust than a 3‐pool model‐based analysis, but the inclusion of an extra pool did not substantially affect the APT effects observed in that study.

The aim of this study was to explore the information a 4‐pool Bloch–McConnell model provides about NOEs in ischemic stroke, contrasting that with the intentionally approximate 3‐pool model. The first objective was to observe the differences between 3‐ and 4‐pool model‐based analysis of NOEs, including their ability to distinguish pathological tissue, and whether these differences are repeatable in higher‐field preclinical data. The second objective was to gain a better understanding of signal origins using a preclinical model of ischemic stroke in order to help inform physiological interpretation of the clinical data. Towards this end a methodological comparison of multipool quantification techniques was undertaken using imaging data from healthy volunteers, a cohort acute stroke patients, and in animals that underwent middle cerebral artery occlusion (MCAO) to induce a focal ischemic lesion.

## 2 THEORY

In multipool model fitting, *z*‐spectra are generated from a set of model parameters which directly describe the underlying physical parameters, such as exchange rate, relaxation times, and proton pool concentrations. Magnetization exchange interactions between bulk water and different solutes are described by separate solute exchange pools. The solute pools transfer saturation to the water pool, on which a detectable signal accumulates. Each pool is described by a set of Bloch equations that are coupled through mass‐conserving exchange rates; the so‐called Bloch–McConnell model.^16^, ^17^ Model fitting for CEST has been done using both least‐squares and Bayesian inference approaches.^18^ Bayesian inference in particular allows the use of parameter priors that are relevant to in vivo data where knowledge of the statistical distribution of tissue parameter values can be incorporated. After fitting multiple pools to the acquired *z*‐spectrum, only a subset are simulated in order to predict the effect arising from a single pool, or a combination thereof, thereby isolating the contribution of each solute pool.^12^, ^14^, ^15^, ^19^

Previously for acute clinical stroke a 3‐pool model has been used by our group, comprising a bulk water pool, an amide pool, and a third pool that attempted to account for both semisolid effects and NOEs.^11^, ^12^, ^13^ The third pool grouped together saturation effects from NOEs and semisolid MT without distinguishing between these two signal sources, and was implemented to reduce confounding of the APT signal. Grouping NOEs and semisolid MT effects into a single pool might be suboptimal given the observed differences in the line width of the semisolid pool signal and the narrower region over which NOEs have been observed,^7^ particularly when seeking to examine changes in these effects in stroke. The 4‐pool model used in the present study expands the 3‐pool model such that semisolid MT and NOEs are described using two separate pools, with the aim of achieving a better signal separation. The NOE pool models a classic Lorentzian effect centred at −3.5 ppm based on observations in the literature, with a single line width spanning composite resonances from −2 to −5 ppm.^3^, ^7^, ^20^, ^21^ For the semisolid MT pool, we have adopted a Lorentzian line shape approximation rather than using a super‐Lorentzian. The semisolid pool thus approximates semisolid effects using a Lorentzian centered at the water resonance, with a very short $T_{2}$ (unobservable on a CEST MRI time scale) that renders a broadband saturation effect.^3^, ^22^ While this approximation limits the quantitative accuracy of semisolid MT measures, it is acceptable for the purpose of isolating NOEs from much broader MT effects. This is because NOEs are evaluated within a relatively narrow frequency range over which the differences between Lorentzian and super‐Lorentzian line shapes are small due to the semisolid pool's short $T_{2}$ and wide line shape.^14^, ^23^ Our construction of the semisolid pool therefore plays more of a correctional role that improves quantitation of CEST effects, rather than being interpreted as an accurate representation of quantitative MT in its own right.

Previously the apparent APT ratio, APTR*, has been defined by Chappell et al.,^18^ and in the same way, the apparent NOE ratio (NOER*) is obtained. NOER* is obtained using a 2‐pool simulation of NOEs and bulk water, ignoring any other pools from the model fitting, and comparing this to a 1‐pool simulation of the water pool; NOER* is the difference between the two simulations measured at −3.5 ppm. This procedure for calculating NOER* is illustrated graphically in Supporting Information S1, section I. Similarly, the apparent semisolid effects ratio, *l*MTR* (*l* denoting *Lorentzian* as opposed to superLorentzian), was obtained by simulating the semisolid and bulk water pools, ignoring any other pools included in the model fitting, and calculating *l*MTR* as the difference between the 1‐ and 2‐pool simulations at 50 ppm. In general, when using a multipool model, the frequency at which the effect of a metabolite pool is quantified is the same as the pool's resonance frequency, as that is where the maximum signal change can be measured. In the case of a symmetric semisolid pool, whose resonance frequency (0 ppm) is identical to the water pool, it is not possible to make a meaningful measurement on resonance as the signal is dominated by water direct saturation. For this reason, the offset of 50 ppm was chosen, similar to that used in classical semisolid MT experiments.^24^

## 3 METHODS

### 3.1 Study details

Six healthy volunteers (median age of 34 years) were recruited and imaged under an agreed technical development protocol approved by the institution's Research Governance Office. These volunteers underwent imaging with four repeated CEST scans at three separate time points (initial, at 24 h and 1 week).

Eighteen patients presenting with acute ischemic stroke were recruited into a prospective observational imaging study following informed consent or agreement from a representative according to research protocols agreed by the UK National Research Ethics Service Committee (references 12/SC/0292 and 13/SC/0362) as previously described.^11^ After exclusions on the grounds of motion corruption, imaging artifacts, and secondary hemorrhage, this left 12 datasets for analysis as described in the original study. The median time from onset was 2 h and 59 min, with a median patient age of 79.5 years, seven female, and a median National Institutes of Health Stroke Scale score at presentation of 11. Individual patient demographics are detailed in Reference 11.

### 3.2 Image acquisition

#### 3.2.1 Human imaging

All participant scans were performed on a 3 T Siemens Verio scanner using a 32‐channel head coil. Each participant underwent a $T_{1}$‐weighted MP‐RAGE structural scan (voxel dimensions $1.8\times 1.8\times 1.0\;mm^{3}$, FOV = 228 mm, TR = 2040 ms, TE = 4.55 ms, TI = 900 ms), diffusion‐weighted imaging (DWI) in three directions (b=0, $b=1000\;s/mm^{2}$), multiple postlabeling delay vessel‐encoded pseudo‐continuous arterial spin labeling perfusion‐weighted imaging,^25^ and single‐slice CEST imaging with voxel dimensions $3\times 3\times 5\;mm^{3}$. Pulsed CEST preparation was performed with 50 Gaussian pulses (20 ms pulse width with a 20 ms interpulse delay) at a flip angle of $184^{\circ }$ to achieve an RMS $B_{1}$ of 0.55μT over the 40 ms period. Crusher gradients were applied between pulses to spoil residual transverse magnetisation. A spin echo EPI readout (TR=5s, TE=23ms, 64×64 matrix size, 6/8 partial Fourier) was performed after the CEST preparation pulses. For patients, the single‐slice CEST imaging plane was localized by a clinician based on the DWI lesion at the time of scanning. An evenly distributed sampling scheme was used up to patient 4, where 32 saturation frequencies were used from −4.5 to 4.5 ppm in steps of 0.3 ppm, and 300 ppm. For patients 5–12, and the healthy volunteers, a semi‐optimal sampling scheme, that had a higher density of points around the amide resonance frequency, was used (−300, −50, −30, −4.1, −3.8, −3.5, −3.2, −2.9, −0.9, −0.6, −0.3, 0.0, 0.3, 0.6, 0.9, 2.9, 3.1, 3.2, 3.3, 3.4, 3.4, 3.5, 3.5, 3.6, 3.6, 3.7, 3.8, 3.9, 4.1, 30, 50, 300ppm).^26^ The total acquisition time for the CEST sequence was 2 min 45 s. A DWI (at 24 h) and/or $T_{2}$‐weighted FLAIR (at 1 week) follow‐up scan was acquired to define tissue outcome.

#### 3.2.2 Preclinical imaging

All animal procedures were approved by the Animal Care and Ethical Review committee of the University of Oxford and the Home Office (UK) and conformed to the Animal (Scientific Procedures) Act 1986 (UK). The preclinical study included six male Sprague‐Dawley rats in which a unilateral ischemic lesion was induced by occluding the origin of the middle cerebral artery with an intraluminal filament introduced through the common carotid artery, as described previously.^27^ MRI was performed on the animals using a 9.4 T Agilent spectrometer with a 72‐mm inner diameter volume transmit coil and four‐channel surface receiver array coils (RAPID Biomedical). Imaging was performed at 1 and 2 h post‐MCAO. Quantitative $T_{1}$ and $T_{2}$ maps were obtained using inversion recovery (TR = 10 s, TE = 8.22 ms, TI = 13.14 to 8000 ms) and spin echo experiments (TR = 10 s, TE = 30 to 160 ms in 10 steps). Cerebral blood flow was measured using multiphase pseudo‐continuous arterial spin labeling (eight phases between $0^{\circ }$ and $315^{\circ }$, tag thickness 6.2 mm, postlabel delay 550 ms),^28^ and the apparent diffusion coefficient (ADC) was measured from DWI in three perpendicular directions with $b=0\;s/mm^{2}$ and $b=1000\;s/mm^{2}$. Apart from the value of $B_{0}$ and the number of saturation frequencies, the preclinical CEST preparation parameters were identical to those used in the human imaging study. Fifty‐one offset frequencies were used (−300, −4.1, −3.8, −3.5, −3.2, −2.9, −2.6, −2.3, −2, −1.7, −1.5, −1.2, −0.9, −0.6, −0.3, 0, 0.3, 0.6, 0.9, 1.2, 1.5, 1.6, 1.7, 1.8, 1.9, 2, 2.1, 2.4, 2.7, 2.9, 3.1, 3.2, 3.3, 3.4, 3.5, 3.6, 3.7, 3.8, 3.9, 4.1, 5, 7, 9.7, 13.5, 19, 26, 37, 51, 72, 100, 300 ppm). Image acquisition following the saturation period was performed using a 10‐slice single‐shot spin echo EPI readout (FOV = 32×32mm, 1 mm slice thickness, TR = 5 s, TE = 27 ms). A CEST acquisition was not available for animal 4 at 2 h post‐MCAO, hence at this time point the total number of animals available for analysis was five.

#### 3.2.3 Phantom preparation and imaging

Seven pH phantoms were prepared to mimic the intracellular environment of naïve mouse brain; refer to the study of Reference 29 for full details of phantom preparation and imaging. Briefly, six female BALB/c mice were terminally anaesthetized, their brains were removed and homogenized, and the intracellular metabolites were extracted by perchloric acid extraction. These metabolites were added to 10% bovine serum albumin (BSA) solutions and titrated to 7 pH values (6.3–8.2), measured using a pH meter. The volume of solution in the phantoms was scaled to match in vivo metabolite concentrations, and the BSA was not cross‐linked in order to avoid introducing semisolid MT effects. MRI was performed on all phantoms simultaneously using a 9.4 T spectrometer (Agilent Technologies) with a volume transmit‐receive coil. The phantoms were imaged at ambient room temperature. Quantitative $T_{1}$ and $T_{2}$ maps were obtained for each phantom. The CEST acquisition comprised 300 Gaussian pulses (26 ms pulse width, $180^{\circ }$ flip angle) at a 50% duty cycle (equivalent to a continuous‐wave $B_{1}$ power of 0.8μT). Eighty‐five saturation frequencies, evenly spaced between ±10ppm were used, and four unsaturated acquisitions at ±500ppm (two of each). Image acquisition following the saturation period was performed using an 8‐shot spin echo EPI readout. The scan parameters were: 38×38mm FOV, 32×32 matrix size, 2 mm slice thickness, TE = 8.22 ms, and TR = 7.85 ms.

### 3.3 Processing

A brief outline of the main processing steps employed in this study is presented below—refer to Supporting Information S1 for full details on each section. Image processing and analysis was performed using the FMRIB Software Library^30^, ^31^ and Python (www.spyder‐ide.org).

#### 3.3.1 Image processing

The skull and nonbrain areas were automatically removed in all of the collected data, and all of the imaging modalities were transferred to structural space. The CEST frequency offsets were motion‐corrected with respect to the unsaturated acquisition. The $T_{1}$ structural data were automatically segmented into cerebrospinal fluid, grey matter (GM), and white matter (WM) (Supporting Information S1, section II).

#### 3.3.2 Region of interest definition

The regions of interest (ROIs) used in this study for stroke patients and animals were: ischemic core, infarct growth, oligaemia, and a contralateral mask. In stroke patients and healthy subjects, analyses were additionally performed within GM and WM masks (Supporting Information S1, section III).

#### 3.3.3 Quantification of CEST effects

A continuous‐wave approximation of the multipool Bloch–McConnell model, including both three and four pools as described in Theory, was fitted to the data^18^, ^32^, ^33^ using the variational Bayes fitting tool BayCEST (www.quantiphyse.org).^12^, ^18^, ^34^ The frequency offset of the CEST images was corrected for $B_{0}$ inhomogeneity via a variable in the model‐fitting algorithm that accounted for water resonance shift. The parameter prior distributions are detailed in Reference 15 for the clinical data, and were used with the following adjustments. For the human data, the NOE pool $T_{2}$ prior mean was 1 ms. For the preclinical data, the $T_{1}$ prior mean for each pool was adjusted assuming proportionality to $B_{0}^{1/3}$:^35^, ^36^ water pool $T_{1}=1.9\;s$, amide pool $T_{1}=1.1\;s$, semisolid pool $T_{1}=1.5\;s$, and NOE pool $T_{1}=1.1\;s$. The amide pool $M_{0}$ prior mean was 90 mM, with a standard deviation (SD) of 20 mM for the human data and 200 mM for the preclinical data. The multipool model‐based analysis is implemented in Quantiphyse, made available for download at https://www.quantiphyse.org.^15^, ^19^, ^37^

In previous studies MT effects arising from semisolid tissue have been quantified using the model‐free conventional MT ratio (MTR) metric, defined as MTR $=\frac{S_{0}-S(50\;\mathrm{ppm})}{S_{0}}$.^24^ This metric was also used in the present study for comparison with *l*MTR*, which is a model‐based equivalent. The $B_{0}$ map obtained from the 4‐pool fit was used to correct this metric for water resonance shift by voxel‐wise interpolation of the acquired z‐spectrum.

The pH phantom data, in which semisolid effects were minimized during the phantom preparation stage, were fitted to a 3‐pool model comprising a water pool, the labile amine proton pool at 2.8 ppm, and a pool centered at −3.5ppm representing NOEs.^29^ The multipool model was fitted to each phantom in sequence. $T_{1}$ and $T_{2}$ scalar priors were used for each phantom, obtained by finding the mean relaxation time from the respective quantitative $T_{1}$ and $T_{2}$ maps, thereby helping to correct for variations in relaxation time.^29^

### 3.4 Analysis

The analysis plan was first to define the repeatability, grey‐white matter contrast $\left(\frac{\mathrm{WM}\;\mathrm{mean}-\mathrm{GM}\;\mathrm{mean}}{\mathrm{WM}\;\mathrm{mean}+\mathrm{GM}\;\mathrm{mean}}\right)$, and correlation characteristics in healthy tissue. The CEST measures were then evaluated across pathological tissue outcomes in patients and rats. The pH dependence of NOER* was assessed in vitro. Finally, a direct comparison between NOER* and the APT equivalent, APTR*, is presented. All analyses were performed in the native space of the data.

#### 3.4.1 Comparison of CEST metrics in patients and stroke models

The CEST metrics were normalised to their mean value in the contralateral hemisphere, producing a relative measure (where a value of 1 indicates the same value as the contralateral ROI). The relative measure accounted for systematic variability between subjects not already controlled for by the quantification technique. The contralateral‐normalized values were averaged by taking the simple mean over subjects. The averaging yielded a group mean for each tissue outcome. In a previous study^15^ we applied the voxel‐weighted mean, but here we have opted for the simple mean and used unweighted tests as this aligns with our uniform assignment of N−1 statistical degrees of freedom for *N* subjects.

#### 3.4.2 Statistical analysis

The statistical tests used were: two‐way ANOVA for assessing subject and time point repeatability in healthy volunteers; Pearson's *r* for correlation between CEST metrics with the corresponding *p* value from a mixed effects analysis; one‐way ANOVA for comparison between pathological ROIs followed by post hoc testing using a two‐tailed unequal variances *t*‐test (internally defines a pooled degrees of freedom); mixed effects analysis for testing differences between the group relative mean of a pathological ROI and the contralateral (assigned a hypothetical value of 1). The number of degrees of freedom used in the statistical tests was based on the number of subjects ($\alpha_{\text{crit}}=0.05$).

## 4 RESULTS

**FIGURE 1:**
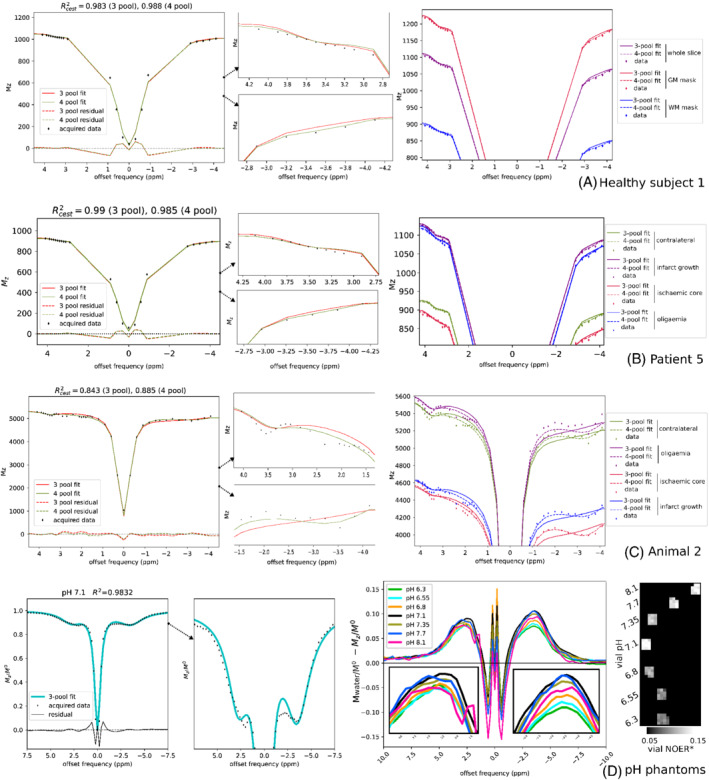
Acquired data and model‐fitted *z*‐spectra shown for various regions of interests in example subjects. The plots report the goodness of fit using the coefficient of determination ($R_{\text{cest}}^{2}$: coefficient of determination evaluated at CEST offsets; approximately 4.5 ppm to 1 ppm upfield and downfield of water). Enlarged views highlight the relevant downfield and upfield CEST regions. (A) Healthy volunteer 1, (B) patient 5, (C) animal 2, and (D) pH 7.1 vial—also showing the acquired data after subtraction of the water baseline, and the vial NOER* maps

**FIGURE 2:**
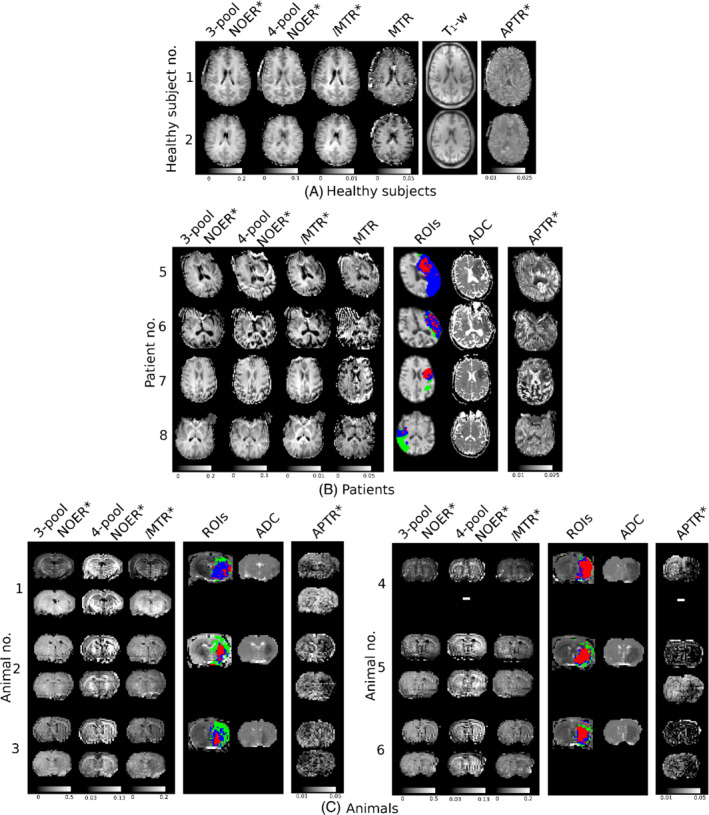
Maps of the CEST metrics in example subjects: (A) healthy volunteers, (B) patients, and (C) animals at 1 h post‐MCAO (top row) and 2 h post‐MCAO (bottom row). Also shown are the apparent diffusion coefficient maps, and the ROIs overlaid on the $T_{1}$‐weighted image (red: ischemic core, blue: infarct growth, green: oligaemia). The APTR* map is included for visual comparison

The acquired spectra and model‐fitted results from example subjects are shown in Figure 1. The corresponding maps of the CEST metrics are shown in Figure 2.

### 4.1 Healthy subjects

The CEST measures generally did not have significant variability between time points or subjects; only 3‐pool NOER* and *l*MTR* had significant subject variability. Each CEST metric exhibited a larger value in white matter compared to grey matter (3‐pool NOER*: 21%, 4‐pool NOER*: 19%, *l*MTR*: 24%, MTR: 28%). Correlation between the CEST metrics was statistically significant, and the strength of correlation was to different degrees. Four‐pool NOER* exhibited the lowest correlation; 0.45 with MTR and 0.53 with *l*MTR*. Three‐pool NOER* had the highest correlation; 0.93 with *l*MTR*, and to a lesser degree with 4‐pool NOER* (0.76). MTR had a comparatively intermediate correlation of 0.65 with both *l*MTR* and 3‐pool NOER*.

### 4.2 Stroke patients

**FIGURE 3:**
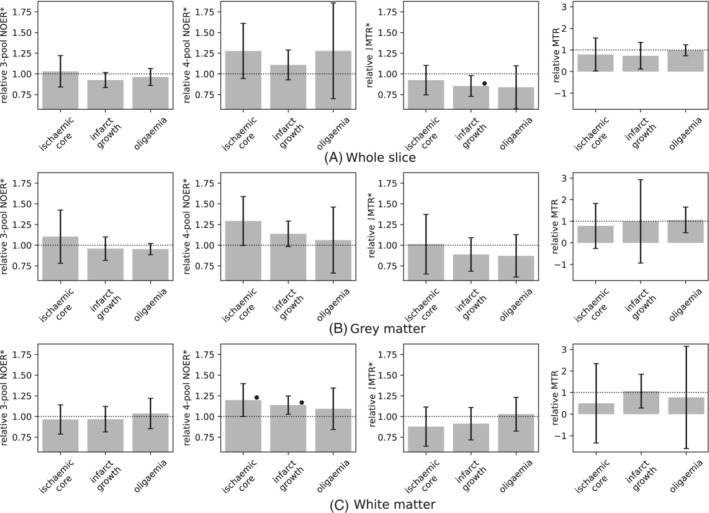
Mean value of each CEST metric in patients, relative to the contralateral hemisphere: (A) whole slice, (B) grey matter voxels, and (C) white matter voxels. Note the wider scale for magnetization transfer ratio. Error bars are the 95% CI. Statistical significance between pathological regions of interests is denoted by a horizontal bar, and by • with respect to the contralateral

The relative (to contralateral tissue) means of each metric are shown in Figure 3 for the pathological ROIs in patients (mean ±95% CI). Significant trends were not observed in grey matter. In white matter, relative 4‐pool NOER* was significantly elevated in the ischemic core (1.20 ± 0.20, p=0.01), and in infarct growth tissue (1.14 ± 0.11, p=0.04). The other measures were not significantly different, except for *l*MTR* being decreased in the infarct growth ROI when the whole slice was considered (*l*MTR*: 0.85 ± 0.13, p=0.01). MTR exhibited a relatively high degree of variance compared to the pool‐based measures and was not included in subsequent analyses.

### 4.3 Preclinical and pH phantom results

**FIGURE 4:**
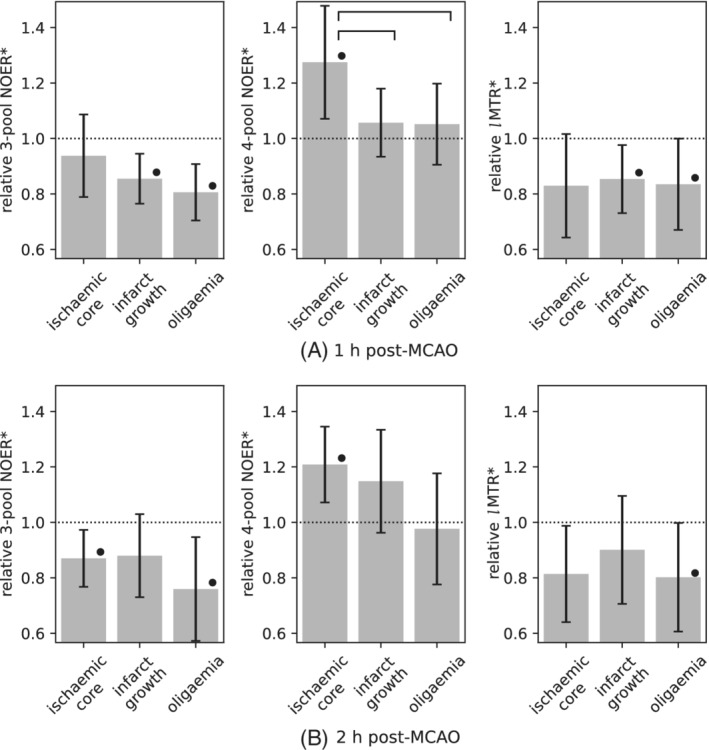
Mean value of model‐based CEST metrics in animals, relative to the contralateral ROI: (A) 1 h post‐MCAO, and (B) 2 h post‐MCAO. Error bars are the 95% CI. Statistical significance between pathological ROIs is denoted by a horizontal bar, and by • with respect to the contralateral

**FIGURE 5:**
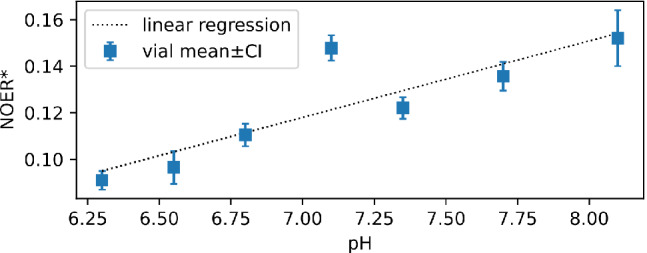
pH dependence of NOER* in naïve brain phantoms titrated to different pH. Error bars are the 95% CI. Black dotted line is the linear regression relationship (0.03× pH −0.11; r=0.87, p=0.01)

The relative means of each metric are shown in Figure 4 for the pathological ROIs in animals. At 1 h post‐MCAO, relative 4‐pool NOER* was significantly elevated (1.27 ± 0.20, p=0.02), and differentiated from both infarct growth (1.06 ± 0.12, P=0.045) and oligaemia (1.05 ± 0.15, P=0.047). At 2 h post‐MCAO, 4‐pool NOER* remained significantly elevated (1.21 ± 0.14, p=0.01). At this time point, the infarct growth mean was higher than at 1 h, but not significantly so (1.15 ± 0.19, p=0.1). Three‐pool NOER* and *l*MTR* were generally decreased, and exhibited similar trends to each other, including significantly decreased values in the oligemia ROI at both time points. In the naïve brain pH phantoms, significant positive correlation between NOER* and pH was observed (r=0.87, p=0.01), shown in Figure 5.

### 4.4 Comparison of NOER* and APTR*

**FIGURE 6:**
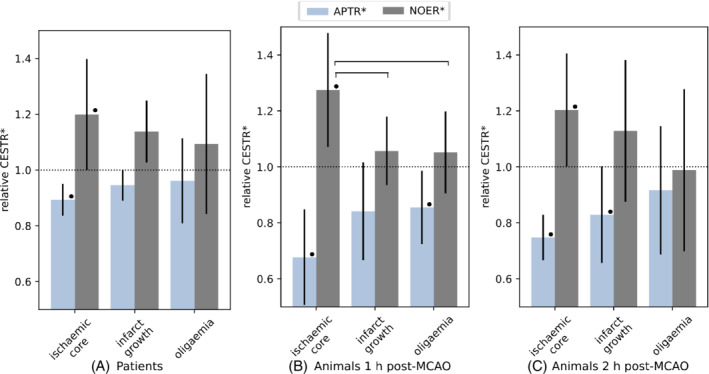
Comparison of (4‐pool) NOER* and APTR* in (A) patients, (B) animals at 1‐h post‐MCAO, and (C) animals at 2 h post‐MCAO. The patient values are from the whole slice for APTR*, and white matter for NOER*. Error bars are the 95% CI. Statistical significance between pathological ROIs is denoted by a horizontal bar, and by • with respect to the contralateral

A comparison of the trends exhibited by (4‐pool) relative APTR* and relative NOER* is presented in Figure 6. APTR* was significantly decreased in patients (0.89 ± 0.06) and in animals (0.67 ± 0.17 at 1 h, 0.75 ± 0.08 at 2 h). In the infarct growth ROI, APTR* was 0.95 ± 0.06 (p=0.07) in patients, 0.84 ± 0.17 (p=0.06) in animals at 1 h, and 0.83 ± 0.17 (p=0.01) at 2 h post‐MCAO. In patients, NOER* lesion contrast was (mean ± SD) 9 ± 16%, and APTR* lesion contrast was −6 ± 35%. In animals, the NOER* and APTR* lesion contrasts were 12 ± 8% and −20 ± 11% respectively.

## 5 DISCUSSION

The aim of this study was to explore the information provided by 4‐pool Bloch–McConnell model of NOEs in ischemic stroke, compared to a 3‐pool approximation. The 4‐pool measure of NOEs exhibited a different association with tissue outcome compared to the 3‐pool approximation. In particular, in the ischemic core, 4‐pool NOER* was elevated in both patient white matter and in animals. In animals an elevated 4‐pool NOER* was observed in infarcted tissue, whether that was the region at 1 h post‐MCAO, or the larger region that includes infarct growth at 2 h post‐MCAO. The observation in the preclinical data was consistent with the clinical data, in that elevated 4‐pool NOER* is indicative of tissue death.

Based on the correlation between 3‐pool NOER* and *l*MTR*, their similar patterns in pathological ROIs, and the relatively poor fit of the NOE peak using three pools, it appears that quantification of NOEs using three pools is strongly biased by a confounding MT effect and does not adequately quantify NOEs as measured using 4‐pool NOER*. The 4‐pool NOE metric utilized a more biologically precise model, with similar trends being observed in the preclinical and patient datasets, and it is reasonable to interpret 4‐pool NOER* as being a metric that better reflects in vivo changes associated with NOEs compared the 3‐pool approximation. While the simpler 3‐pool model did not capture changes in NOEs as clearly, the study of Reference 15 on APT quantification found that three pools were better at discerning infarct growth, precisely because the amide pool retained some NOE‐based contribution. This could point to a trade‐off between optimizing for contrast of tissue sought for clinical intervention, and biophysically more accurate modeling of CEST effects.

The preclinical results show that the 4‐pool model captures some temporal changes in the evolution of infarct growth. Furthermore, the temporal change were relatively large using NOER* as compared to APTR*, which seems to be a differentiating feature of the NOE compared to the APT effect. Stroke injury initially occurs over minutes to hours and is followed by secondary injury from hours to days, followed by subsequent complications. CEST signal dynamics over these timescales could be mirroring different compensatory mechanisms of the brain associated with delayed cellular injury. The results provide motivation for exploring NOEs using serial imaging.

A manner in which NOER* and APTR* bear similarity is their consistency between healthy subjects and time points, which has been reported previously for APTR*,^15^ and assessed for NOER* in the present study. The repeatable nature of a CEST metric is one requirement for clinical imaging. Another requirement in clinical imaging contexts is that a CEST metric maximises lesion contrast. By extension, the metric would preferably minimise grey‐white matter contrast and thereby exhibit similar patterns of change in response to ischemia. In this respect, NOER* may be less favorable because it has a prominent white matter contribution compared to that reported for APTR* (1%–2% depending on how contrast is defined),^15^ although the patterns of change in grey and white matter appear to be similar. The main way in which APTR* excels is lesion contrast; while absolute contrast of APTR* was smaller in patients, it was much more consistent than NOER* (and preclinically, both larger *and* more consistent). Another way of looking at this is by reference to the contralateral‐normalized measures; the absolute effect size of relative NOER* in patients was larger than relative APTR*, but disproportionately more variable.

Although APTR* generally exhibited more desirable contrast features, an appeal of CEST MRI is the potential complementarity of different contrasts that can be obtained in a single acquisition. In this respect, it is worth noting the graded trend of NOER* as a function of tissue injury; 4‐pool NOER* exhibited the highest increase in the ischemic core, followed by infarct growth, and was closest to normal (the contralateral ROI) in the oligaemic tissue, which is mildly underperfused and not at risk of infarction. This observation on NOER* inversely mirrors the graded variation in APTR*, and can be seen on both the patient and animal datasets (Figures 5C and 6A). These divergent patterns as a function of the severity of injury indicate a type of complementarity with respect to pathology between the NOE and APT. The results highlight the relevance of analyzing the association of NOEs with regions of the brain remote from the DWI lesion.

Changes in NOER* are concurrent with the decrease of APT effect in the ischemic core,^4^, ^15^, ^38^ which raises the question of whether NOEs are sensitive to pH, as the APT effect is generally assumed to be. NOEs can depend on pH when magnetization of aliphatic protons is first dipolar‐coupled to labile protons of amide side‐groups on the same molecule, before finally undergoing chemical exchange with bulk water protons.^3^ In the study of Reference 10 the build‐up of NOEs on non‐chemically‐exchanging lipids was similar to that obtained using BSA protein phantoms that permit chemical exchange, suggesting that NOEs may manifest through pathways of both intermolecular dipolar coupling and pH‐dependent chemical exchange.^7^ An indirect dependence of NOEs on pH can be realized through changes in protein conformation that reduce the number of dipolar coupling sites available,^9^, ^39^ although this might require a pH change larger than that caused by ischemia, and sufficient for effecting a high level of protein chaos. Experimentally, there have been differing results as to whether NOEs are pH‐sensitive.^3^, ^7^ The study of Reference 3 on pH phantoms found that NOEs were insensitive to pH, however the insensitivity to pH might have been an artifact of the subtraction technique used to quantify NOEs, where there was a cancellation of pH‐related changes in signal.^7^ In our study, NOER* exhibited significant correlation with pH. A comparable study to the one conducted herein is the study of Reference 7 which analyzed the pH dependence of NOEs in 10% BSA phantoms at 7 T using the Lorentzian difference analysis technique, reporting a positive pH dependence of NOEs. Therefore, in an in vitro setting where metabolite concentration is controlled for and pH is decreased, NOEs appear to change in the same (downwards) direction as base‐catalyzed amides.

In vivo it might not be possible to attribute changes in NOEs solely to pH, as decreases in both pH and protein concentration have been reported in samples from ischemic lesions.^40^ Rather than observing a reduced NOE signal as might be expected under circumstances of lower pH and lower protein concentration, we have instead seen an elevated 4‐pool NOER* signal in the ischemic core, consistent with some other preclinical studies.^41^, ^42^, ^43^ This nonintuitive finding cannot be fully explained in terms of the known mechanisms for NOEs, as it does not appear to concord with the positive correlative with pH demonstrated in phantoms and the expected decrease in pH and sites for CEST interactions.

In ischemia both acidosis and alkalosis have been described. The observation of elevated NOER* might be related to alkalotic shift of tissue pH, observed in the subacute stroke study of Reference 44, which the authors attributed to active compensatory mechanisms after acute cerebral ischemia because of still‐viable cell populations in the ischemic tissue. Alternatively, referring to the nonlinear feature in pH phantom data, the locally negative pH trend imparted by the pH 7.1 vial extending to the pH 7.35 data point could explain the decrease in NOER* if healthy and acidotic tissue are bracketed in this pH range. This range of pH variance between healthy and infarcted tissue seems plausible given that, in preclinical models, intracellular pH is estimated at 7.0−7.25 depending on the technique used, with a theoretical maximum pH change of −0.9 units under physiologically reproducible ischemic conditions.^45^ NOEs in the ischemic core appear to be affected by competing processes, and alludes to a complex array of mechanisms at play in the early stages of ischemic stroke, inviting further experiments to explain the phenomenon.

In healthy subjects the larger white matter NOER* value compared to grey matter was consistent with previous studies on NOEs.^3^, ^7^, ^42^, ^46^ The relatively narrow line width of NOE spectra, characteristic of mobile species with short $T_{2}$, has led to the suggestion that NOEs may originate from mobile lipid content found in the white matter myelin sheath.^10^ At higher field strengths, studies of NOEs in vivo reveal several discrete resonances over the range from −2 to −5ppm, indicating a composite nature of NOE resonance.^7^, ^20^ In the present study, however, NOEs were modeled as a single pool rather than as multiple distinct resonances. This simplification was used because the discrete resonances significantly overlap at the lower $B_{0}$ field strength used in this study, and there were insufficient data points to justify more than a single exchange pool, the aim of which was to capture an ensemble measure of the NOEs.

In the 4‐pool model, semisolid MT effects are modelled as a symmetric phenomenon, with NOEs imparting an upfield asymmetry the *z*‐spectrum. In early CEST studies it was thought that *z*‐spectra were symmetrical about the water resonance in the absence of metabolite exchanges, and a broad featureless spectrum upfield of the water resonance was assumed, and used as a reference in spectral asymmetry analyses. However, asymmetry of the upfield spectra, particularly at lower RF saturation powers, indicated the presence of strong saturation effects in the aliphatic region of the spectrum. The cause of asymmetry was initially attributed to resonance mismatch between the bulk water and semisolid pools such that semisolid macromolecules had a positive chemical shift with respect to the water signal.^21^, ^47^, ^48^, ^49^, ^50^ On the other hand, the broad spectral distribution of semisolid effects, on the order of tens of ppm,^3^, ^21^ suggests that the spectrum could be symmetric. Some earlier studies have attributed this asymmetry to the aliphatic protons of a mixture of mobile to relatively mobile peptides, proteins, lipids, and metabolites, which have resonances located around −3.5ppm, ranging from −2 to −5ppm.^21^ Upfield exchange processes related to mobile macromolecules,^21^, ^51^ or NOEs, are now thought to be the cause of *z*‐spectrum asymmetry,^3^, ^7^, ^8^, ^21^, ^51^ and we made this assumption in our choice of a symmetric MT pool.

Semisolid effects originate from immobile lipids which facilitate rapid magnetization transfer via spin diffusion, having a much shorter $T_{2}$ compared to the surrounding water and a correspondingly broad line width.^52^, ^53^, ^54^ Semisolid MT has typically been used in identifying white matter disease which is marked by demyelination of nerve axons in white matter, yielding lower magnetization transfer ratios.^52^ In a previous study on semisolid MT, saturation was much decreased in the ischemic core imaged at 1 month, possibly reflecting a decrease in the concentration of cell membrane‐bound proteins as a consequence of the cell death and membrane degradation which occurs following ischemic stroke.^24^ In terms of measuring MT effects, MTR has previously been applied to proton density‐weighted images acquired using a low flip angle and a long repetition time.^55^, ^56^ In our study a relatively large flip angle was used which was optimised for imaging APT CEST effects, and this may explain why our results using conventional MTR appeared to be insensitive to changes in pathological tissue.^57^

We observed that *l*MTR* was decreased in all pathological ROIs relative to the contralateral normal tissue. There is some ambiguity in interpreting this observation, as the way in which we modeled semisolid effects is approximate compared to modeling used in the quantitative MT literature.^58^ The multipool formalism describes each pool using a single transverse relaxation time, which analytically yields a Lorentzian absorption line shape.^59^ The Lorentzian line shape is suitable for mobile populations which can be described using a single $T_{2}$. The semisolid MT pool, however, is composed of immobilized proteins and large lipids that are restricted in motion, for which this assumption may not be a good approximation if accurate quantitation of semisolid MT is sought.^60^, ^61^ Super‐Lorentzian and Gaussian line shapes have been found to provide a better fit for tissue, with the former giving optimum results in vivo and is used in model‐based MT studies.^22^, ^23^, ^52^, ^62^ In the present study we have adopted a Lorentzian line shape for the semisolid MT pool rather than using a super‐Lorentzian. While this approximation limits the quantitative accuracy of semisolid MT measures, it is acceptable for the purpose of isolating NOEs from much broader MT effects. This is because NOEs are evaluated within a relatively narrow frequency range over which the differences between Lorentzian and super‐Lorentzian line shapes are small due to the semisolid pool's short $T_{2}$ and wide line shape.^14^, ^23^ Our construction of the semisolid pool therefore plays more of a correctional role that improves quantitation of CEST effects, rather than being interpreted as an accurate representation of quantitative MT in its own right. The model fitting function used in this study has been developed further by Smith et al.^58^ to include a super‐Lorentzian line shape, rendering it suitable for making quantitative MT measures simultaneously with CEST in future studies.

**FIGURE 7:**
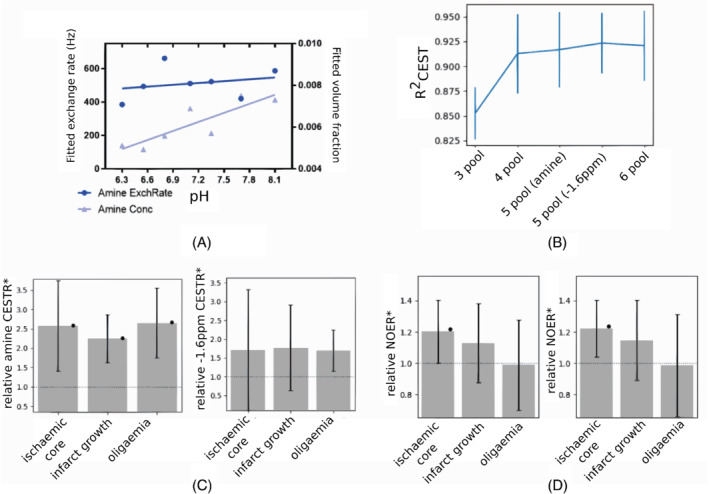
Tertiary analyses exploring some of the limits of multipool modeling of our data. (A) Fitted exchange rate and volume fraction from multipool analysis of naïve rat brain pH phantoms. (B) Goodness‐of‐fit as a function of number of pools, applied to the animal data. Error bars are the standard deviation across 11 rat datasets. (C) Five‐pool model of AmineR* at 1 h, and 5‐pool measure of effect at −1.6 ppm at 2 h. (D) NOER* using four pools, and using five pools that include the −1.6 ppm pool

We conducted a number of brief tertiary analyses (Figure 7) to illustrate some of the main limitations of multipool fitting on our data, assessing specificity for individual physical variables and the effects of adding more pools. The NOER* metric, and *CEST*R* in general, is a nonrate‐specific measure that seeks to explain overall changes driven by any combination of exchange rate and volume fraction. The effects of a CEST pool's volume fraction and exchange rate have very similar effect on the appearance of the *z*‐spectrum, and it was for this reason that the APTR* metric was originally developed.^18^ When using only one $B_{1}$ power for saturation, as was the case for the clinical data available in this study, the physical model does not reliably isolate concentration effects from exchange rate effects as it uses the volume fraction variable to explain changes that are actually due to pH (exchange rate), meaning we do not have specificity for explaining those individual physical parameters (Figure 7A). The individual parameter maps do not purely represent exchange rate or volume fraction, and thus do not add meaningfully to the interpretation in isolation. The fitted NOE volume fraction and exchange rate can only be meaningfully interpreted when combined into a single quantity, NOER*.

Various model‐based CEST studies have sought to additionally quantify amines at 2 ppm and the NOE at −1.6 ppm using five to six pools in total.^42^, ^43^, ^63^ The NOE at −1.6 ppm has been reported to exhibit a significant decrease immediately after stroke^42^, ^43^ and hypointensity in tumours.^63^, ^64^, ^65^, ^66^ The peak at −1.6 ppm has been closely associated with NOE‐mediated interactions between choline head groups of membrane phospholipids, which have a resonance frequency around −1.6 ppm, and water protons.^63^ Adding further pools to our modeling introduces a risk of over‐fitting to the clinical dataset, particularly because these peaks are close to water resonance and sampling density is low. On the preclinical data, the largest improvement in goodness‐of‐fit is obtained by going from a 3‐pool model to a 4‐pool mode (Figure 7B). Adding the amine pool and the pool at −1.6 ppm (either separately as five pools, or collectively as six pools) seems to provide a small additional improvement. However, a good fit does not necessarily translate to adequate quantification of the amine or −1.6 ppm features. For example, in the 5‐pool AmineR* results (Figure 7C), the values do not seem plausible as they are very high (more than 10 times the APT effect size), and also variable. The amount of variability (coefficient of variation; CoV = SD ÷ mean) in the contralateral was 380%, compared with only 50% for APTR* and NOER*. For completeness, we also repeated the analysis of this 5‐pool model with a stronger assumption on the amine in‐vivo mean concentration and SD, and whilst the results had less variability (CoV: 140%), it was still large and AmineR* was eight times bigger than the APTR*. A similar trend was observed when using a 5‐pool model that includes the peak at −1.6 ppm; the CoV was 200%. The 6‐pool results (not shown) were similarly highly variable. These results suggest the data are insufficient for extracting a consistent trend for amines and at −1.6 ppm. One reason for the inconsistent results might be the relatively low $B_{1}$ power employed in this study, 0.55μT, which is not optimal for observing saturation effects at −1.6 ppm. $B_{1}$ dependence studies suggest using a power of 1μT at 9.4T,^65^, ^66^ and other studies of NOEs at −1.6 ppm have also used this power.^42^, ^63^ The relatively few sampling points in the vicinity of −1.6 ppm would have also contributed to the lack of differentiation; only one sampling point was located within the peak's full‐width half‐maximum (−1.8 to −1.4 ppm), whereas studies that have detected a significant difference in stroke lesions have generally acquired three offsets.^42^, ^64^, ^65^ NOEs at −1.6 ppm are particularly vulnerable to under‐fitting owing to their proximity to the water peak, and this may explain why such a peak has not been reported in lower‐field clinical studies.^42^

We also looked at how the inclusion of pool at −1.6 ppm affects NOER*. It is possible that although we cannot adequately quantify effects at −1.6 ppm, inclusion of the additional pool will nevertheless absorb some variability that is not as well explained by the main NOE pool alone, yielding an improvement in NOER*. Figure 7D shows that there is negligible effect on relative NOER* when a 5‐pool model is used that includes the pool at −1.6 ppm.

## 6 CONCLUSION

The 4‐pool measure of NOEs provided information on tissue outcome that was different from the 3‐pool approximation. The 3‐pool measure of NOEs was strongly weighted by semisolid MT effects. The 4‐pool NOE metric, by virtue of employing a more biophysically accurate model, may be better at capturing in vivo changes in NOEs. NOEs were elevated in the ischemic core, but given that both pH and protein concentration have previously been reported to decrease in infarcted tissue, the observations still cannot be fully explained in terms of known NOE mechanisms. NOEs appear to be affected by competing processes, and alludes to a complex array of mechanisms at play in the early stages of the disease. Further investigation of NOEs is needed in order to better‐understand the physiological origins of these observations and their potential as complementary imaging biomarkers in acute stroke.

## CONFLICT OF INTEREST

PJ is the Editor‐in‐Chief of Magnetic Resonance in Medicine. In line with COPE guidelines, he recused himself from all involvement in the review process of this paper, which was handled by an Associate Editor. He and the other authors have no access to the identity of the reviewers. MAC, PJ, and TOK receive royalties for commercial licensing of the FMRIB Software Library.

## Supporting information

Supporting Information

## Data Availability

For enquiries regarding the preclinical data, contact NS (https://nicola.sibson@oncology.ox.ac.uk) and for enquiries regarding the clinical data, contact JK (https://james.kennedy@rdm.ox.ac.uk).
