# Supplementary material for: Quantitative chemical exchange saturation transfer imaging of nuclear overhauser effects in acute ischemic stroke
Source: Magn Reson Med. 2022 Mar 7;88(1):341–56. doi: 10.1002/mrm.29187 (PMC9314583; doi:10.1002/mrm.29187)
Supplement: Supplementary file 1 — Supporting Information [file MRM-88-341-s001.pdf]

Supporting Information S1

I. Obtaining *CEST*R* metrics

Figure 1 provides a high-level outline of how 4-pool NOER* is obtained, based on the original definition of APTR* for amide proton transfer [1]. The CEST data are fitted to a 4-pool model which accounts for a water pool, amides, NOEs, and semisolid MT effects. The output of the model fitting process is a number of model-fitted parameters corresponding to different variables in the physical model. In order to derive NOER*, which is an isolated quantitative measure of NOEs, only two model-fitted parameters are used: NOE pool relative concentration, NOE exchange rate. A 1-pool model is used to simulate water-only saturation, and a 2-pool model is used to simulate the contribution of the NOE pool. NOER* is defined as the difference between the two simulated line shapes at the NOE resonance frequency, -3.5 ppm. An equivalent procedure is used to calculate lMTR*, where semisolid pool parameters are used at the simulation stage; 3-pool NOER*, where initial model fitting is done using only 3 pools; and APTR*.

II. Image processing

The BET tool in the FMRIB Software Library (FSL) package [3] was used to remove the skull and non-brain areas in all of the collected data. All of the imaging modalities were transferred to the T_1 space; within time point image registration was performed using FSL FLIRT, and, across time point image registration using FNIRT, both available in the FSL package [4]–[6]. The different CEST frequency offsets were motion-corrected using MCFLIRT, which applied linear co-registration to achieve alignment with the unsaturated acquisition [4]. MCFLIRT non-default options were: *stages=4* which internally uses sinc interpolation for more robust optimisation, and *sinc-final* to apply the final output transformations using sinc interpolation. The quantified CEST effects were transformed to the T_1 image space using FLIRT. The T_1 structural data were segmented using FAST into cerebrospinal fluid, GM and WM.

III. ROI definitions in native space

In stroke patients, infarct at presentation was defined using semi-automated delineation of ADC below an externally validated threshold of 620×10^{-6} mm^2/s [7]. Final infarct was manually defined preferentially on the 1 week FLAIR image, or, if not available, the $b = 1000$ DWI at 24 hours [8]. The mask representing perfusion deficit was generated using a threshold approach where voxels with a cerebral blood flow (CBF) threshold of less than 20 ml/100g/min were identified and clustered, and then used as a guide for manual delineation by an expert clinician (GH) [9]. The ROIs used in this study were:

- Ischaemic core: within both presenting and final infarct definitions.
- Infarct growth: within the final infarct, but not within the presenting infarct.
- Oligaemia: tissue present in the perfusion deficit but not the final infarct.

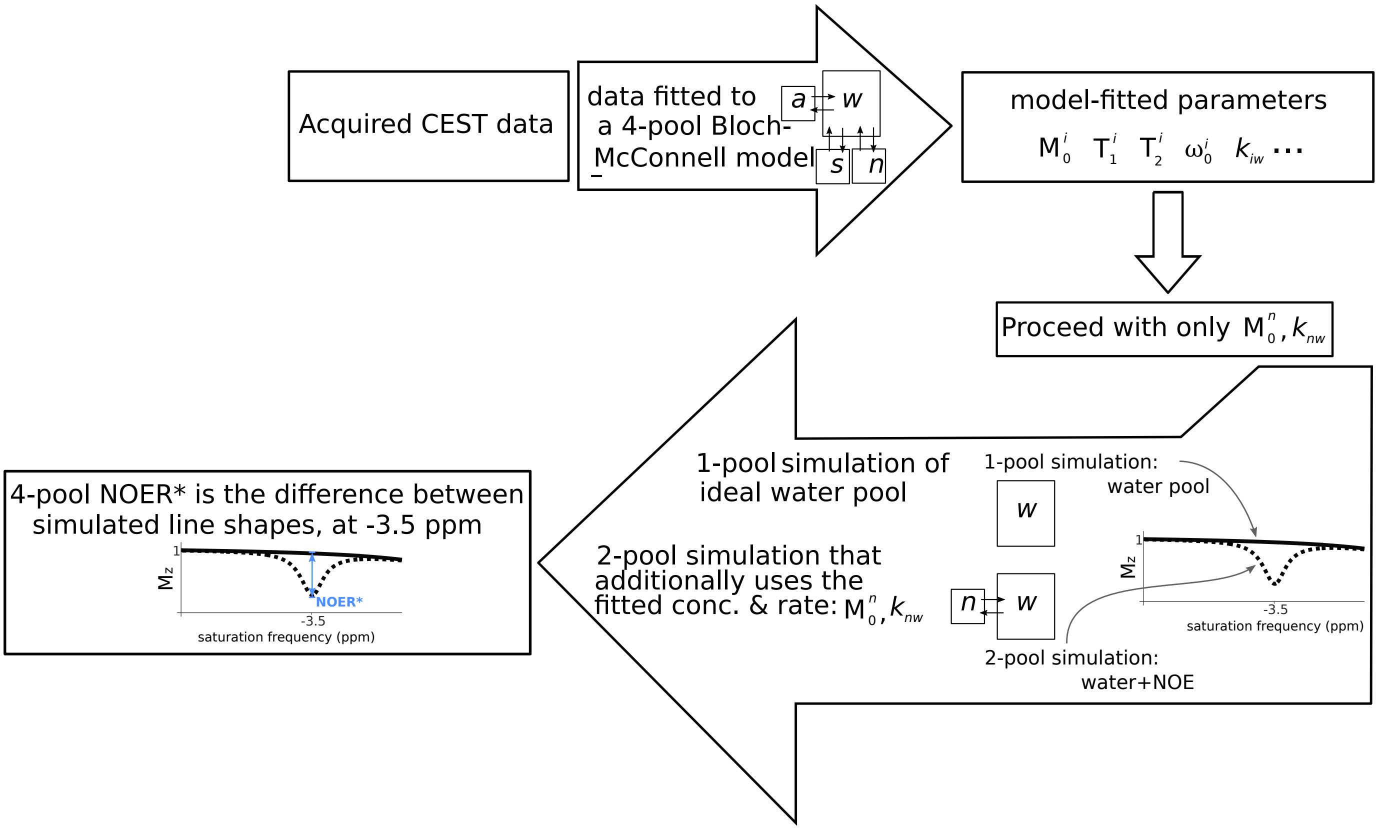

Fig. 1: Flow diagram illustrating how 4-pool NOER* is obtained, based on how ref. [1] defined APTR*. The four pools are: water w, amide a, semisolid MT s, and NOEs n. For calculating 3-pool NOER*, s and n are replaced with a single pool approximation. Figure adapted with permission from ref. [2].

- Mirrored contralateral mask: a contralateral ROI was obtained for each patient by non-linearly registering the union of the pathological masks to standard MNI152 space, reflection in the sagittal plane, and transforming back to CEST space.

These ROI definitions are in keeping with those used in ref. [9] but have been updated to improve ROI fidelity with tissue fate [8]. A whole slice mask, a grey matter mask, and a white matter mask, were defined as follows for the healthy subjects and stroke patients. Grey matter and white matter masks were first generated from partial volume (PV) estimates using FSL FAST [10] on the presenting T_1-weighted scan and the images were transformed to the resolution of the CEST images. Thresholds were applied to create healthy subject masks in the data space:

- Whole slice mask: voxels with a GM ∪ WM PV threshold of 50%.
- GM mask: voxels with a GM PV threshold of 70%.
- WM mask: voxels with a WM PV threshold of 90%.

For the preclinical data, pathological ROIs representing ischaemic core, infarct growth, oligaemic, and contralateral tissue, were defined automatically based on the differences between the 1 h and 2 h post-MCAO scans, where the ADC and CBF maps were thresholded using the values defined in

the study of ref. [9]. All brain voxels were included in the analyses of animal data (no PV threshold defined).

References

[1] M. A. Chappell, M. J. Donahue, Y. K. Tee, A. A. Khrapitchev, N. R. Sibson, P. Jezzard, and S. J. Payne, “Quantitative Bayesian model-based analysis of amide proton transfer MRI,” *Magn Reson Med*, vol. 70, no. 2, pp. 556–567, Aug 2013.

[2] Y. Msayib, “Quantifying impaired metabolism following acute ischaemic stroke using CEST MRI,” Ph.D. dissertation, University of Oxford, Oxford, UK, 2017.

[3] S. M. Smith, “Fast robust automated brain extraction,” *Hum Brain Mapp*, vol. 17, no. 3, pp. 143–155, Nov 2002.

[4] M. Jenkinson, P. Bannister, M. Brady, and S. Smith, “Improved optimization for the robust and accurate linear registration and motion correction of brain images,” *Neuroimage*, vol. 17, no. 2, pp. 825–841, Oct 2002.

[5] M. Jenkinson, C. F. Beckmann, T. E. Behrens, M. W. Woolrich, and S. M. Smith, “FSL,” *Neuroimage*, vol. 62, no. 2, pp. 782–790, Aug 2012.

[6] M. Jenkinson and S. Smith, “A global optimisation method for robust affine registration of brain images,” *Med Image Anal*, vol. 5, no. 2, pp. 143–156, Jun 2001.

[7] A. Purushotham, B. C. Campbell, M. Straka, M. Mlynash, J. M. Olivot, R. Bammer, S. M. Kemp, G. W. Albers, and M. G. Lansberg, “Apparent diffusion coefficient threshold for delineation of ischemic core,” *Int J Stroke*, vol. 10, no. 3, pp. 348–353, Apr 2015.

[8] G. W. J. Harston, D. Minks, F. Sheerin, S. J. Payne, M. Chappell, P. Jezzard, M. Jenkinson, and J. Kennedy, “Optimizing image registration and infarct definition in stroke research,” *Annals of Clinical and Translational Neurology*, vol. 4, no. 3, pp. 166–174, 2017.

[9] G. W. J. Harston, Y. K. Tee, N. Blockley, T. W. Okell, S. Thandeswaran, G. Shaya, F. Sheerin, M. Cellerini, S. Payne, P. Jezzard, M. Chappell, and J. Kennedy, “Identifying the ischaemic penumbra using pH-weighted magnetic resonance imaging,” *Brain*, vol. 138, no. Pt 1, pp. 36–42, Jan 2015.

[10] Y. Zhang, M. Brady, and S. Smith, “Segmentation of brain MR images through a hidden Markov random field model and the expectation-maximization algorithm,” *IEEE Trans Med Imaging*, vol. 20, no. 1, pp. 45–57, Jan 2001.

List of Figures

1 Flow diagram illustrating how 4-pool NOER* is obtained, based on how ref. [1] defined APTR*. The four pools are: water w, amide a, semisolid MT s, and NOEs n. For calculating 3-pool NOER*, s and n are replaced with a single pool approximation. Figure adapted with permission from ref. [2]. 2
